# Musculoskeletal Injuries in Master Swimmers: A National Survey in Turkey

**DOI:** 10.7759/cureus.8421

**Published:** 2020-06-03

**Authors:** Halis Atilla, Mutlu Akdogan, Alper Öztürk, Mehmet Baris Ertan, Ozkan Kose

**Affiliations:** 1 Orthopaedics and Traumatology, Dıskapı Yıldırım Beyazıt Education and Research Hospital, Ankara, TUR; 2 Orthopaedics and Traumatology, University of Health Sciences, Antalya Education and Research Hospital, Antalya, TUR; 3 Orthopaedics, University of Health Sciences, Antalya Education and Research Hospital, Antalya, TUR

**Keywords:** sports medicine, swimming, athletic injuries, musculoskeletal pain

## Abstract

Background

This study aimed to determine the frequency of musculoskeletal injuries in master swimmers in Turkey.

Methods

A questionnaire was formed and distributed to all master swimmers registered with the Turkish Swimming Federation. The collected data included age, sex, the age to start swimming (SAS), weekly training time (WTT), weekly training distance (WTD), any painful episode that lasted more than 10 days in any of the body regions within last one year, any confirmed diagnosis of musculoskeletal disease by a physician, and history of musculoskeletal surgical operation. The descriptive data were presented, and multiple comparisons were made according to demographic characteristics.

Results

There were 88 male swimmers with a mean age of 47.1±13.2 years (range, 26-89 years). Of the 88 athletes, 27 (30.7%) had no pain in daily activities, and 61 (69.3%) reported pain in at least one region, with a total of 118 pain zones reported. The shoulder was the most common painful body region (n:37, 42.0%), followed by the lower back (n: 24, 27.3%), neck (n: 19, 21.6%), back (n:12, 13.6%), and knee (n:9, 10.2%). The mean age, SAS, WTT, WTD, and distribution of stroke preference were similar in subjects with or without reported pain and diagnosis (p>0.005). Any painful body region and diagnosis were equally distributed in all swimming styles (p>0.05). The most common surgeries were lumbar disc disease (16.7%) and meniscectomy (16.7%).

Conclusion

Compared to the findings in the current literature, master swimmers do not have as many musculoskeletal problems as their younger counterparts. The problems seen in master swimmers are lower but similar to those in competitive elite swimmers. From the musculoskeletal health perspective, swimming is safe for the master age group. Swimming can be safely offered to elderly patients who underwent even musculoskeletal surgery.

## Introduction

With a better understanding of the favorable effects of sport on general health, individuals with or without an active sports background engage in sports suitable for their health conditions at various ages. Maintaining muscle mass is crucial for a healthy body and a longer life. Exercise such as swimming builds muscle strength and power and functional ability in the activities of daily living in older adults [1]. Swimming provides an excellent non-weight-bearing alternative to exercise for people in all age groups. Unlike jogging or running, swimming does not overload the musculoskeletal system while it provides cardiovascular conditioning. This is highly advantageous for older people, especially those with arthritis in their joints. Therefore, many older people choose to swim as a recreational activity.

However, swimming is not entirely free from injuries. Overuse injuries are especially common [2]. In several previous studies, swimming injuries have been well-studied but there are few epidemiological studies on master swimmers, a relatively older group of athletes [3-4]. This study aimed to determine the frequency of musculoskeletal injuries in master swimmers involved in competitive swimming in Turkey and to investigate the relationship of musculoskeletal problems with stroke preference and other subject characteristics.

## Materials and methods

This study was a prospective observational study conducted on licensed master swimmers over 25 years old. A questionnaire was formed and distributed to all master swimmers registered with the Turkish Swimming Federation to collect the data. Subjects who were unwilling to participate in the study and subjects who are not active swimmers for the last three months were excluded. This study was carried out following the ethical standards laid down in the 1964 Declaration of Helsinki and its later amendments, and informed consent was obtained from all participants. The local ethics committee approved the study protocol. A total of 115 master swimmers responded and completed the questionnaire. Due to incomplete data entry, 13 were excluded from the study. As 88 males and 14 females remained, the female swimmers were excluded due to the insufficient number. Finally, 88 male swimmers were included in the study for analysis.

The questionnaire included age, the age to start swimming (SAS), weekly training time (WTT), weekly training distance (WTD), any painful episode lasting more than 10 days in any of the body regions within the last one year, any confirmed diagnosis of musculoskeletal disease by a physician, and history of musculoskeletal surgical operation. The questionnaire was administered to the participants either through a face-to-face interview or a phone call.

Continuous variables were presented as mean and standard deviation, and categorical variables were stated as count and percentage. The Kolmogorov-Smirnov test was used to determine whether the data were distributed normally. A comparative analysis between independent groups was performed using the Mann-Whitney U test, independent sample student's t-test, and chi-square test. A value of p < 0.05 was accepted as statistically significant.

## Results

There were 88 male swimmers with a mean age of 47.1±13.2 years (range, 26-89 years). Since participants competed with more than one swimming style, a total of 149 different styles have been reported, including freestyle (n:73), breaststroke (n:30), backstroke (n:22), and butterfly style (n:24). The mean age to start swimming was 22.3±16.9 years (range, 5-60 years). The mean training time per week was 5.4±2.5 hours (range, 0-12 hours). The mean training distance per week was 10082.9±7601.8 meters (range, 0-50.000 meters). The mean age to start swimming was younger in the butterfly style (14.4±13.2 versus 25.3±17.3 p=0.007). Of the 88 athletes, 27 (30.7%) had no pain in daily activities and 61 (69.3%) reported pain in at least one region, with a total of 118 pain zones reported. The shoulder was the most common painful body region (n:37, 42.0%), followed by the lower back (n: 24, 27.3%), neck (n: 19, 21.6%), back (n:12, 13.6%), and knee (n:9, 10.2%) (Table 1).

**Table 1:** Distribution of painful body regions among participants

Pain Location		n (%)
1	Shoulder	37 (42.0%)
2	Lower Back	24 (27.3%)
3	Neck	19 (21.6%)
4	Back	12 (13.6%)
5	Knee	9 (10.2%)
6	Elbow	8 (9.1%)
7	Hip	5 (5.7%)
8	Hand/Wrist	2 (2.3%)
9	Foot and Ankle	2 (2.3%)

The mean age, SAS, WTT, WTD, and swimming styles were similar in subjects with or without reported pain (Table 2). Any painful body region was equally distributed in all swimming styles (p>0.05). 

**Table 2:** Comparison of subjects with at least one painful body region versus subjects without self-reported pain SAS: Age to start swimming, WTT: Weekly training time, WTD: Weekly training distance

Variable	Subjects With Pain (n:61)	Subjects Without Pain (n:27)	Significance
Age (years ± SD)	45.8±12.6	50.2±14.3	0.152
SAS (years ± SD)	20.5±15.7	26.5±19.1	0.127
WTT (hours per week ±SD)	5.6±2.4	4.9±2.8	0.220
WTD (meters per week ±SD)	10483.6±6584.0	9177.7±9597.0	0.461
Freestyle (% within group)	78.7%	92.6%	0.094
Breaststroke (% within group)	36.1%	29.6%	0.369
Backstroke (% within group)	19.7%	37.0%	0.073
Butterfly (% within group)	23.0%	37.0%	0.134

A previous confirmed musculoskeletal diagnosis was reported by 34 (38.6%) swimmers and 54 (61.4%) had no diagnosis of musculoskeletal condition or injury. The most common diagnosis was related to upper extremity problems (35.6%) (Table 3).

**Table 3:** Distribution of confirmed diagnosis according to the body region ACL: Anterior cruciate ligament

Confirmed Musculoskeletal Diagnosis		n (%)	Cumulative (%)
Upper Extremity	Subacromial impingement	5 (11.1%)	35.6%
	Biceps tendinitis	3 (6.6 %)	
	Partial rotator cuff rupture	2 (4.4%)	
	Glenohumeral osteoarthritis	1 (2.2%)	
	Glenohumeral dislocation	1 (2.2%)	
	Acromioclavicular separation	1 (2.2%)	
	Proximal humeral fracture	1 (2.2%)	
	Forearm fracture	1 (2.2%)	
	Carpal tunnel	1 (2.2%)	
Spine	Lumbar disc disease	8 (17.7%)	26.7%
	Cervical disc disease	1 (2.2%)	
	Scoliosis	1 (2.2%)	
	Spondylolisthesis	1 (2.2%)	
	Ankylosing spondylitis	1 (2.2%)	
Knee	ACL rupture	3 (6.6 %)	24.5%
	Knee osteoarthritis	3 (6.6 %)	
	Meniscal lesion	3 (6.6 %)	
	Acute patellar dislocation	1 (2.2%)	
	Knee contusion	1 (2.2%)	
	Iliotibial band syndrome	1 (2.2%)	
Ankle	Ankle sprain	2 (4.4%)	8.9 %
	Lateral malleolus fracture	1 (2.2%)	
	Achilles tendinitis	1 (2.2%)	
Hip	Femoral neck fracture	1 (2.2%)	2.2%
Total		45 (100%)	100

The mean age, SAS, WTT, WTD, and distribution of stroke preference were similar in subjects with or without confirmed diagnosis (p>0.05 for all variables). The presence of a confirmed musculoskeletal diagnosis was equally distributed in all swimming styles (p>0.05). A history of musculoskeletal system surgery was reported by 15 (17%) of the study participants. A total of 18 operations were reported by 15 swimmers. The most common operations were arthroscopic meniscus surgery (n:3, 16.7%), lumbar discectomy (n:3, 16.7%), and anterior cruciate ligament reconstruction (n:2, 11.1%). Other surgeries were fibular fracture fixation, forearm fracture fixation, bilateral total knee replacement, Bankart repair, carpal tunnel release, femoral neck fracture fixation, humerus fracture fixation, ankle ligamentous repair, and anterior tibial tendon repair.

## Discussion

The results of this study showed that there was no significant relationship between the age of the master swimmer with pain, diagnosis, and a history of surgery. Baker et al. reported that swimming-related injury prevalence increased significantly with advancing age [5]. However, no such relationship has been found in other master athletes [6]. In the current study, comparisons were made between those who started swimming in childhood and those who started swimming as adults with respect to WTT, WTL, pain, diagnosis, surgery, and stroke. A difference between the groups was only determined with respect to stroke. The butterfly stroke rate of those who started swimming after the age of 18 years was significantly low. Relatively difficult strokes such as butterflies can be made by those who have been swimming since childhood. It was observed that butterfly stroke is not preferred by swimmers, who began swimming in adulthood.

Upper extremity, knee, and spine problems are often seen in elite athletes due to high repetitive movements during normal swimming [2]. In elite athletes, the injury rate in 1000 hours of exercise has been reported to be 4 in men and 3.78 in women elite swimmers. Shoulder injuries have been reported to be the most common injuries in swimmers, with a prevalence of 40%-91% [7-8]. This is due to the fact that, unlike in many other sports, the arms are responsible for the explosive movement for the propulsion of the body.

Shoulder injuries are often manifested by rotator cuff impingement, biceps tendinitis, and shoulder instability. In a study of 80 elite swimmers, 91% of elite swimmers reported shoulder pain. While 84% of those athletes had positive impingement findings, 69% of 52 swimmers with MRI had supraspinatus tendinopathy findings [8]. In the current study, the highest pain was reported in the shoulder region (42.0%), and the most commonly diagnosed musculoskeletal pathology was shoulder lesions. However, when previous surgeries were examined, with the exception of one patient who had a Bankart lesion repair due to instability, no other patients had undergone shoulder surgery. The relatively low number of shoulder operations as compared to musculoskeletal pathology and pain could be attributed to master swimmers not returning to swimming after these operations as much as after other region surgeries.

Krüger et al. administered a questionnaire to 282 master swimmers, and the incidence of shoulder pain over three years was 62.4% [9]. Statistically significant relationships were determined between shoulder pain and the risk factors of osteoporosis, the volume of training, and competitiveness. However, no significant association was found between age, gender, body mass index (BMI), swimming stroke, strength training, and the incidence of shoulder pain. In a study by Tate et al., 64 master swimmers were analyzed, and there was reported to be a 19.4% rate of shoulder pain and disability [10]. Stocker et al. compared two age groups of collegiate and master swimmers and reported similar percentages of shoulder pain despite the shorter distances and lower intensity of training associated with the master group [11]. In the current study, shoulder pain was the most common pain, and there was no relationship between training duration and training length.

The knee is the second most frequently reported area of pain for competing swimmers. As with other swimming injuries, the primary factor is overuse [12]. In a study of competitive swimmers, 34% reported knee problems [13]. In the current study, the rate of knee pain in master swimmers was found to be 10.2%. In the literature, knee problems are more common in breaststroke swimmers [14]. Freestyle swimmers are at a lower risk of knee injuries [15]. Medial knee pain has been reported by up to 75% of those swimming breaststrokes [16]. Increased varus and valgus overload and rapid extension in the knee are thought to be responsible. There is increased tension in the medial compartment and increased compression in the lateral compartment. Breaststroke has been shown to be responsible for this knee pain with a thickened inflamed medial plica [17].

In the current study, when the pathological diagnoses of the knee joint are examined, 24.5% of all the pathological diagnoses were in the knee region. The swimming styles of individuals with a diagnosis of meniscus lesions alone were examined, and it was seen that this group only swam freestyle. Of the master swimmers who had undergone meniscectomy, both breaststroke and freestyle were seen. Although freestyle is seen as the standard swimming stroke of all master swimmers, it can be concluded that master swimmers can continue to swim breaststroke even after meniscectomy surgery.

Compared to elite athletes in the literature, it was observed that breaststroke swimming master athletes had fewer knee symptoms. Nichols stated that symptoms from a degenerative meniscus tear in an older athlete might be exacerbated by breaststroke swimming due to the forceful rotatory and valgus loads on the knee [18]. In the current study, the master swimmers with a diagnosis of meniscus lesion did not report swimming breaststroke.

Lower back problems, including lumbar discs, are common in swimmers. Various levels of the degenerative disc have been shown in 68% of elite swimmers and 29% of recreational swimmers [19]. Exercise frequency, duration, and distance have been reported to increase lumbar spine disc degeneration in competing swimmers. In the present study, no relationship was found between exercise duration and distance and any musculoskeletal condition. Kaneoka et al. showed no change in disc disease according to gender or swimming style [19]. In contrast, Nyksa et al. stated that, especially in breaststroke and butterfly, the excessive hyperextension of the lower back forces the posterior lower back structures to cause spondylolysis and spondylolisthesis [20]. In particular, low back pain was found in 33.3% of butterfly and 22.2% of breaststroke swimmers [21]. In the current study, master swimmers reported pain in the spine region as in the lower back (27.3%), neck (21.6.1%), and back (13.6%), respectively. The lumbar region was the second most common region of pain after the shoulder. Lumbar disc disease was ranked second after shoulder lesions in terms of diagnosed pathology (17.7%). A history of lumbar disc surgery was reported by 16.7% of the current study participants. Those with low back pain and lumbar disc disease were found to swim all strokes, and those with operated lumbar disc disease only swam freestyle. It was observed that the swimmers with a lumbar pathology of a serious enough level to require surgery did not prefer breaststroke and butterfly styles. However, those with low back pain or un-operated lumbar disc disease could swim all styles, including butterfly and breaststroke. As the amount of competition for master swimmers is relatively low compared to elite athletes, with the exception of those with serious low back problems requiring surgery, master swimmers with lumbar disc disease or lumbar pain could continue to swim butterfly and breaststroke. Spondylolisthesis was reported in one patient, and this patient could only swim breaststroke.

Female swimmers have been shown to experience significantly more knee, lower back, neck, shoulder, hip, and foot problems [22]. Since the questionnaires of only male patients were examined, no gender comparison could be performed in the current study.

There are few studies in the literature of the musculoskeletal injuries and diagnoses of master swimmers, but there are comparisons of elite athletes and other master athletes.

Studies on musculoskeletal injuries in master swimmers are limited. To the best of our knowledge, this is the first study on the musculoskeletal injuries of master swimmers in Turkey. Only one study was reported related to the epidemiological data of swimmers in Turkey, which included 38 competitive male swimmers, and the prevalence of musculoskeletal problems was determined as 60.52% [21]. In studies of master athletes other than master swimmers, injuries common to master athletes include rotator cuff injuries, Achilles tendinopathies, and meniscal tears [23].

Ganse et al. stated that healthy master athletes have a lower risk of injuries and illnesses during competition than young elite athletes [24]. In a study by Walsh et al., the results suggested that healthy master athletes have a low risk of injury that does not increase with age or performance [25]. In the current study, it was also found that master swimmers had a lower incidence of injury as compared to reports of elite swimmers in literature.

There are some limitations of this study, primarily that only male master swimmers were included. This observational study is not free of selection bias, thus casual relationships cannot be definitively drawn. Personal details that can lead to musculoskeletal problems, such as BMI or osteoporosis, were not studied. Nevertheless, this study is one of the very few studies on the epidemiology of injuries in master swimmers.

## Conclusions

Master swimmers appear to have fewer musculoskeletal problems than their younger counterparts reported in the current literature. Master swimmers who have undergone surgery for musculoskeletal problems can continue swimming. As swimming improves physical performance without any impact on the weight-bearing joints, it provides a safe opportunity to improve physical condition. The problems seen in master swimmers are similar to but at a lower rate than those in competitive, elite swimmers. For elderly patients, even those who have undergone musculoskeletal surgery, but not shoulder surgery, swimming can be safely recommended.
